# Transition zone: the sequential assembly of its components parallels its dual role in basal body anchoring and ciliary function

**DOI:** 10.1186/2046-2530-4-S1-P26

**Published:** 2015-07-13

**Authors:** A Aubusson-Fleury, M Lemullois, H Bengueddach, S Abdallah, L Shi, J Cohen, AM Tassin, F Koll

**Affiliations:** 1 CGM UPR3404, CNRS, Gif Sur Yvette, France

## Objectives

The assembly of cilia can be followed step by step in the unicellular *Paramecium*, thanks to its predictable spatio-temporal pattern of basal body (BB) duplication and ciliary growth. In order to dissect the process of transition zone (TZ) assembly, we compared the behaviour of several proteins.

## Methods

Combination of EM, immunocytochemistry, protein tagging and RNAi knockdowns.

## Results

Two proteins, FOR20 and OFD1, present at the level of the terminal TZ plate in ciliated BB, are recruited early during BB assembly, are required for building the nascent BB tip where a cap is detected and are necessary for BB anchoring. After anchoring and before ciliation, a structure similar to the TZ, defined as the pro-TZ, is detected at the BB tip. At that stage, OFD1 and FOR20 are detected at the level of the proximal pro-TZ plate. In contrast, two other proteins involved in the ciliary barrier function, MKS2, and in the axonemal building, IFT57, are detected only at ciliated BB and recruited at time of ciliation in correlation with the extension/maturation of the pro-TZ into TZ. The depletion of these proteins does not affect BB docking.

## Conclusion

1. The assembly of the transition zone proceeds stepwise (building of a cap on nascent BB; differentiation of a pro-transition zone on anchored non ciliated BB; maturation of the transition zone during ciliation).

2. These steps correlate with the recruitment of proteins required for BB anchoring and ciliary growth/function respectively, highlighting the dual role of the TZ in the process of ciliation.

